# Effects of Socioeconomic and Geographic Factors on Outcomes in Ewing Sarcoma: A National Cancer Database Review

**DOI:** 10.7759/cureus.25525

**Published:** 2022-05-31

**Authors:** Kevin M McMahon, Thomas Nilles-Melchert, Vincent Eaton, Peter T Silberstein

**Affiliations:** 1 Medical School, Creighton University School of Medicine, Omaha, USA; 2 Oncology, Creighton University School of Medicine, Omaha, USA

**Keywords:** national cancer database, mortality, geographic factors, socioeconomic factors, race, ewing sarcoma

## Abstract

Purpose: Ewing sarcoma is a primary malignant bone tumor that manifests predominantly in the proximal long bones and pelvis and traditionally presents with nonspecific symptoms. This tumor preferentially affects children and young adults, occurring most often in patients of European descent. The most important established prognostic factor is the presence of metastasis at the time of diagnosis followed by primary site, size of the primary neoplasm, patient age, and lactate dehydrogenase (LDH) levels. To the authors’ knowledge, this is the first study focused on the effects of socioeconomic and geographic factors on overall survival in Ewings sarcoma.

Methods: A total of 3,920 patients diagnosed with Ewing sarcoma were identified in the National Cancer Database (NCDB) using the International Classification of Diseases for Oncology, Third Edition (ICD-O-3) code 9260. Of these, 3,238 met the inclusion criteria and were analyzed. Descriptive statistics, Kaplan-Meier survival curves, and Cox regression tables were all performed using IBM SPSS Statistics for Windows, Version 27.0 (Released 2020; IBM Corp., Armonk, New York, United States).

Results: Univariate analysis showed greater mortality for patients of increasing age at the time of diagnosis, at two, five, and 10 years of follow-up, Black race patients at two years, Medicare insurance status at two years, urban or rural residence at two and 10 years, more advanced tumor stage at two and five years, and patients with a comorbidity score of ≥2 at two years. Multivariate analysis showed greater mortality at two years with increasing age, Black race, uninsured status, urban or rural residence, and increasing tumor stage. Mortality also increased for patients at five years of follow-up in patients with increasing age or more advanced tumor stage.

Conclusion: Patient mortality in the first two years after diagnosis is increased for patients of the Black race, those living in urban or rural areas, and for patients that are uninsured or using Medicare as their primary payor at the time of diagnosis. To improve patient outcomes, clinicians should recognize and address not only the unique biology of patients but also their unique challenges in access to healthcare. Patients and providers should work to elicit changes on an individual and community level to improve their personal health and the health of those around them.

## Introduction

Ewing sarcoma is the second most common primary bone tumor (after osteosarcoma) affecting pediatric and young adult populations [1,2]. It has a peak incidence at 10-20 years of age, occurs most often in patients of European descent, and is more common in males than females (1.6:1.0 ratio) [1,3]. It manifests primarily in the pelvis and proximal long bones but may also manifest in other bony or extraosseous sites [1]. Extraosseous manifestations are much more common in adults than in pediatric patients [1]. Presenting symptoms are often nonspecific, with the most common being localized pain and swelling that may be intermittent and of variable intensity [1,2]. Other symptoms include a palpable mass and pathological fractures, or if the tumor is in an advanced stage, fevers, night sweats, fatigue, and/or weight loss [1]. Imaging often shows “moth-eaten” lytic bone lesions, a Codman triangle, and/or an “onion peel” appearance [1]. Diagnosis is primarily via histology and molecular analysis of tissue samples with a definitive diagnosis based on finding a t(11;22) (q24;q12) translocation shown in 85-90% of cases [1].

The most important established prognostic factor is metastasis at the time of diagnosis with the lungs, bones, and bone marrow being the most common sites [1]. Five-year survival is often less than 30% with metastasis vs 70% without metastasis [1]. Other important prognostic factors include primary site, size of the primary lesion, patient age, and lactate dehydrogenase (LDH) levels. More proximal tumors, larger tumors, age greater than 18 years, and increased LDH levels are associated with a poorer prognosis [1].

Treatment of Ewing sarcoma generally begins with multidrug chemotherapy that is followed by surgical excision or resection, and then additional chemotherapy to eliminate any remaining malignant cells [3-5]. Radiation may be used for postsurgical management or in place of surgery if there would be an unacceptable loss of function with surgery, such as in the case of bowel or bladder dysfunction [4,5].

The purpose of this study is to analyze the effects of geographic and socioeconomic factors on patient survival in Ewing sarcoma. To the authors’ knowledge, this is the first study conducted through the National Cancer Database (NCDB) assessing the effects of socioeconomic and geographic factors on outcomes in Ewing sarcoma in the United States.

This article was previously presented as a meeting abstract at the 2022 Medical Student Orthopedic Symposium on April 10, 2022.

## Materials and methods

A retrospective review of Ewing sarcoma cases was conducted using the most recent data available through the NCDB [6]. The NCDB was formed to improve patient outcomes and now contains approximately 70% of all new patients in the United States diagnosed with cancer. Professional registrars enter individual level de-identified data from accredited Commission on Cancer (CoC) facilities (American College of Surgeons certified hospitals) [7]. The 2017 Participant User File (PUF) for bone and joint tumors was obtained from the NCDB and contains all data used in this study. The 2017 PUF contains data collected on patients diagnosed between 2004 and 2017.

Patients with Ewing sarcoma were identified using the International Classification of Diseases for Oncology, Third Edition (ICD-O-3) histology code 9260. Initially, 3,920 patients were identified, but this number was reduced to 3,238 when patients with more than one malignancy, unknown vital status, unknown income, and/or unknown rural/urban status were filtered out. For the multivariate analysis, all patients with any missing variables were excluded resulting in a total of 1,267 patients. 

All data were analyzed using IBM SPSS Statistics for Windows, Version 27.0 (Released 2020; IBM Corp., Armonk, New York, United States). Data were analyzed for frequencies of age, sex, race, Spanish/Hispanic ethnicity, insurance status, median income (2008-2012), urban/rural status, percent no high school completion (2008-2012), great circle distance (distance traveled between residence and diagnosing facility), tumor stage, and Charlson-Deyo score. Means were also calculated for age, great circle distance, and follow-up duration. Charlson-Deyo score is a weighted score derived from the sum of scores of identified comorbid conditions. Conditions such as myocardial infarction, dementia, diabetes, renal disease, or acquired immunodeficiency syndrome (AIDS) are scored from one to six points. The weighted Charlson-Deyo score ranges from zero to three with zero meaning no comorbidities, one meaning single comorbidity, two meaning two comorbidities or a single comorbidity with a weight of two (such as renal disease), and three meaning the patient has significant comorbidities [7].

Age data was represented as seven categories (0-10, 11-20, 21-30, 31-40, 41-50, 51-60, 61+). Race was reported as three categories (White, Black, other/unknown) as all racial categories except White and Black represented less than 1% of cases (<30 cases). Spanish/Hispanic origins were divided into four classes (non-Spanish/Hispanic, Mexican, Spanish/Hispanic/Latino not otherwise specified (NOS), and other) as all other NCDB identifications represented less than 1% of cases. Great circle distance was reported in four categories (0-24 miles, 25-49 miles, 50-99 miles, and 100+ miles). All other factors were broken down by categories present within the NCDB. Median income, percent no high school completion, urban/rural status, and great-circle distance were collected by the NCDB based on the ZIP code in which the patient resided at the time of diagnosis. Survival trends were calculated using Kaplan-Meier survival curves for the effect of individual variables on overall survival at two, five, and ten years of follow-up. Cox proportional multivariate models were performed to estimate the independent effects of patient characteristics on mortality at two, five, and ten years of follow-up.

## Results

This study identified a mean age at diagnosis of 20.63 ± 13.23 years with a median age of 17 years and a peak incidence at 16 years. Patient travel distance had a mean of 61.62 miles and a median of 22.10 miles with a range of 0-4912 miles. The mean follow-up duration for patients was 53.38 ± 42.17 months with a median of 41.40 months and a range of 0-182 months. Frequencies for age, sex, race, Spanish/Hispanic ethnicity, insurance status, median income (2008-2012), urban/rural status, percent no high school completion (2008-2012), great circle distance, tumor stage, and Charlson-Deyo score are listed in Table 1.

**Table 1:** Kaplan-Meier Univariate Survival and Patient Characteristics at Two, Five, and Ten Years NOS: Not Otherwise Specified

	Survival % (Deaths)						
Variable (census)	2-year	P-Value	5-year	P-value	10-year	P-value	Mean Survival (Months)
Age		<0.001		0.003		<0.001	
0-10 (564)	91.67 (47)		82.62 (98)		80.14 (112)		141.38
11-20 (1495)	82.61 (260)		67.63 (484)		63.75 (542)		114.81
21-30 (655)	69.92 (197)		54.5 (298)		51.60 (317)		86.23
31-40 (231)	65.80 (79)		54.55 (105)		50.22 (115)		80.98
41-50 (150)	65.33 (52)		50.67 (74)		49.33 (76)		87.38
51-60 (83)	56.63 (36)		43.37 (47)		40.96 (49)		67.6
61+ (60)	40.00 (36)		30.00 (42)		23.33 (46)		37.0
Sex		0.210		0.525		0.283	
Male (2022)	76.61 (473)		63.11 (746)		59.55 (818)		103.93
Female (1216)	80.76 (234)		66.94 (402)		63.90 (439)		114.65
Race		<0.001		0.491		0.690	
White (2884)	78.85 (610)		64.81 (1015)		61.34 (1115)		109.76
Black (115)	60.87 (45)		50.43 (57)		49.57 (58)		76.55
Other (239)	78.24 (52)		68.20 (76)		64.85 (84)		101.35
Ethnicity		0.460		0.164		0.848	
Non-Hispanic (2665)	77.97 (587)		64.24 (953)		60.83 (1044)		109.01
Mexican (55)	85.45 (8)		74.45 (14)		70.91 (16)		83.55
Spanish/Hispanic/Latino NOS (288)	80.90 (55)		65.97 (98)		63.19 (106)		98.81
Other (230)	75.22 (57)		63.91 (83)		60.43 (91)		100.09
Insurance Status		<0.001		0.659		0.789	
Uninsured (149)	68.46 (47)		59.73 (60)		56.38 (65)		85.56
Private Insurance (2138)	80.40 (419)		67.07 (704)		63.80 (774)		111.27
Medicaid (699)	77.68 (156)		62.37 (263)		58.80 (288)		106.25
Medicare (78)	50.00 (39)		34.62 (51)		34.61 (54)		47.55
Other Governmental (70)	74.29 (18)		58.57 (29)		55.71 (31)		90.65
Unknown (104)	73.08 (28)		60.58 (41)		56.73 (45)		100.83
Median Income		0.045		0.089		0.004	
< $38,000 (480)	77.71 (107)		62.92 (178)		57.08 (206)		94.65
$38,000-47,999 (755)	75.23 (187)		60.79 (296)		56.95 (325)		99.88
$48,000-62,999 (846)	77.07 (194)		64.18 (303)		61.70 (324)		108.54
>$63,000 (1157)	81.07 (219)		67.93 (371)		65.25 (402)		115.01
Percent No High School Completion		0.678		0.119		0.314	
>21% (566)	75.62 (138)		61.66 (217)		57.24 (242)		98.22
13-20.9% (802)	78.05 (176)		63.09 (296)		59.23 (327)		105.01
7-12.9% (1000)	78.10 (219)		65.20 (348)		62.10 (379)		108.04
<7% (870)	0.80 (174)		67.01 (287)		64.48 (309)		114.14
Urban/Rural Status		0.018		0.096		0.010	
Metro (2705)	78.82 (573)		65.84 (924)		62.81 (1006)		111.99
Urban (481)	75.47 (119)		58.84 (198)		53.64 (223)		92.66
Rural (52)	71.15 (15)		50.00 (26)		46.15 (28)		77.96
Great-Circle Distance		0.893		0.024		0.722	
0-24 miles (1742)	78.01 (383)		65.61 (599)		62.11 (660)		111.12
25-49 miles (609)	76.85 (141)		64.04 (219)		61.25 (236)		107.39
50-99 miles (436)	80.73 (84)		66.06 (148)		61.93 (166)		104.87
100+ miles (451)	78.05 (99)		59.65 (182)		56.76 (195)		93.02
Tumor Stage		<0.001		<0.001		0.098	
Stage I (377)	91.78 (31)		79.58 (77)		75.60 (92)		131.68
Stage II (935)	87.38 (118)		76.58 (219)		72.94 (253)		126.50
Stage III (88)	82.72 (21)		56.82 (38)		53.41 (41)		92.14
Stage IV (886)	61.06 (345)		42.55 (509)		39.84 (533)		66.69
Unknown (952)	79.83 (192)		67.96 (305)		64.50 (338)		115.79
Charlson-Deyo Score (Comorbidities)		0.039		0.178		0.621	
0 (3058)	78.45 (659)		64.88 (1074)		61.54 (1176)		109.73
1 (138)	77.54 (31)		62.32 (52)		57.97 (58)		98.58
2 (32)	59.38 (13)		43.75 (18)		40.63 (19)		50.60
3 (10)	60.00 (4)		60.00 (4)		60.00 (4)		74.03

Univariate analysis

**Figure 1:**
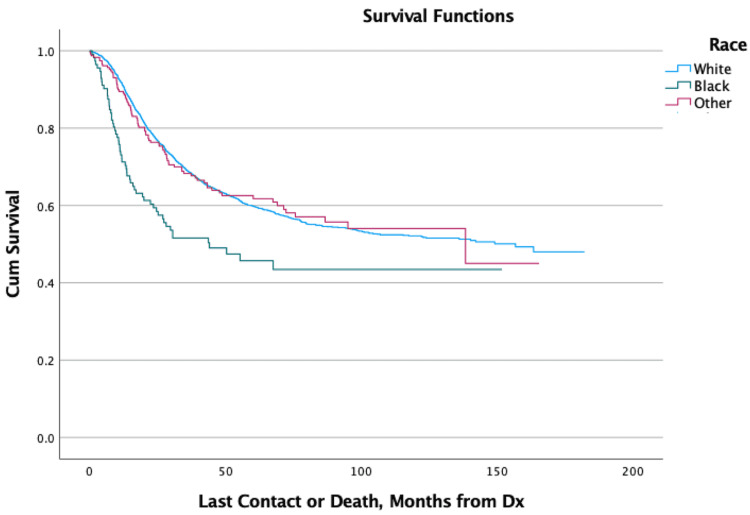
Overall Survival for Patients by Race Dx: Diagnosis; Cum: Cumulative

**Figure 2:**
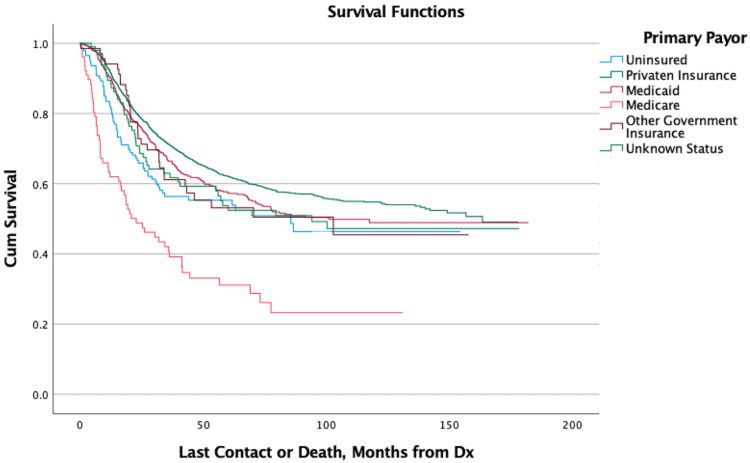
Overall Survival for Patients by Insurance Status Dx: Diagnosis; Cum: Cumulative

**Figure 3:**
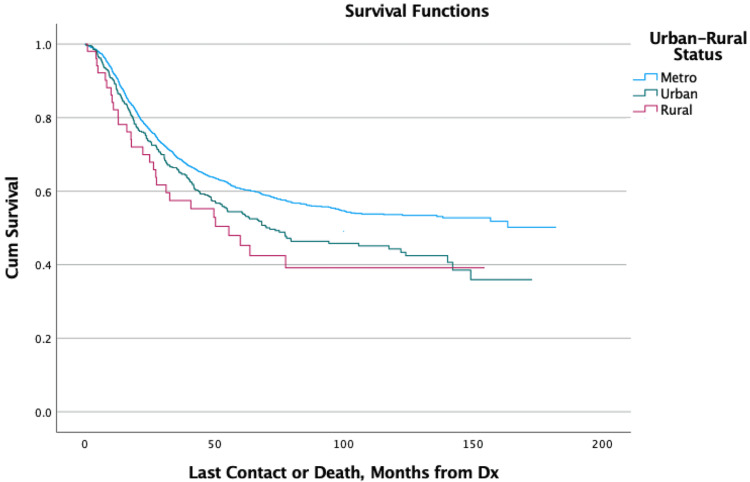
Overall Survival for Patients by Urban-Rural Status Dx: Diagnosis; Cum: Cumulative

Kaplan-Meier curves showed that patients aged 0-10 had the greatest overall survival at two, five, and ten years of follow-up compared to all other age groups, all of which had increasing mortality with each successive decade of life. Patients of Black race showed increased mortality within the first two years of diagnosis but afterward showed similar survivability to other races (Figure 1). Patients that were uninsured or utilizing Medicare as their primary payor at the time of diagnosis showed increased mortality within the first two years (Figure 2). Patients living in urban or rural areas showed increased mortality compared to those living in metropolitan areas (Figure 3). Patients with more advanced stage tumors showed greater mortality at two and five years. Patients with higher Charlson-Deyo scores also showed greater mortality for increasing scores within the first two years. Analysis of median income showed increased mortality with lower income levels. Analysis of travel distance showed increased mortality at five years for patients traveling more than 100 miles between their place of residence and the treatment facility. No statistically significant relationships were found between mortality and sex, ethnicity, or percent high-school completion (Table 1).

Multivariate analysis

The results of the multivariate cox proportional analysis are listed in Table 2. They showed that compared to patients aged 0-10 years, patients aged ≥21 years had increased mortality at two years, patients aged 11-50 years had increased mortality at five years, and patients aged 31-40 years had increased mortality at two, five, and ten years. Compared to White race patients, patients of Black race had increased mortality at two years. Compared to uninsured patients, patients with private insurance, Medicaid, or other governmental insurance had lower mortality at two years. Compared to patients living in metropolitan areas, patients in urban or rural areas had increased mortality at two years. Compared to patients with stage I tumors, patients with tumors of stage III, IV, or unknown stage had increased mortality at two and five years. Compared to patients without any comorbidities, patients with a Charlson-Deyo score of one had increased mortality at two years. No statistically significant relationships between mortality and sex, travel distance, or percent high school completion were observed.

**Table 2:** Cox Regression Multivariate Survival and Patient Characteristics at Two, Five, and Ten Years *Reference: The reference variable is the variable against which all variables are compared to calculate hazard ratios. The odds of the event (death) occurring for other variables are calculated relative to the reference variable. **No Hazard Ratio could be calculated due to the absence of deaths for the variable in this stratum NOS: Not Otherwise Specified

	Hazard Ratio (95% CI)	P-value	Hazard Ratio (95% CI)	P-value	Hazard Ratio (95% CI)	P-Value
Variable	2-year		5-year		10-year	
Age						
0-10	*Reference		*Reference		*Reference	
11-20	1.307 (0.951-1.797)	0.099	1.645 (1.207-2.243)	0.002	1.729 (0.951-3.146)	0.073
21-30	1.488 (1.068-2.073)	0.019	2.016 (1.425-2.854)	<0.001	1.962 (0.963-4.000)	0..064
31-40	2.059 (1.417-2.992)	<0.001	1.636 (1.003-2.668)	0.048	3.184 (1.370-7.402)	0.007
41-50	2.047 (1.363-3.076)	<0.001	2.597 (1.540-4.380)	<0.001	0.866 (0.186-4.033)	0.855
51-60	3.965 (2.512-6.260)	<0.001	1.417 (0.724-2.770)	0.309	1.576 (0.335-7.399)	0.565
61+	6.212 (3.662-10.538)	<0.001	2.329 (0.852-6.362)	0.099	8.990 (2.347-34.432)	0.001
Sex						
Male	*Reference		*Reference		*Reference	
Female	1.095 (0.928-1.293)	0.283	0.979 (0.803-1.195)	0.838	0.805 (0.531-1.216)	0.301
Race						
White	*Reference		*Reference		*Reference	
Black	1.808 (1.312-2.492)	<0.001	0.975 (0.538-1.768)	0.934	0.335 (0.045-2.515)	0.301
Other	1.251 (0.925-1.693)	0.146	0.756 (0.487-1.172)	0.211	0.941 (0.435-2.033)	0.877
Ethnicity						
Non-Hispanic	*Reference		*Reference		*Reference	
Mexican	0.767 (0.373-1.576)	0.470	0.739 (0.319-1.710)	0.480	0.862 (0.194-3.828)	0.845
Spanish/Hispanic/Latino NOS	0.979 (0.725-1.322)	0.889	1.267 (0.892-1.799)	0.187	0.693 (0.313-1.534)	0.366
Other	0.796 (0.597-1.063)	0.122	1.432 (0.931-2.201)	0.102	0.839 (0.392-1.798)	0.653
Insurance Status						
Uninsured	*Reference		*Reference		*Reference	
Private Insurance	0.678 (0.496-0.926)	0.015	1.213 (0.682-2.158)	0.512	0.965 (0.353-2.640)	0.945
Medicaid	0.687 (0.490-0.964)	0.030	1.253 (0.691-2.272)	0.458	0.909 (0.329-2.518)	0.855
Medicare	0.831 (0.503-1.374)	0.471	1.167 (0.502-2.712)	0.719	1.718 (0.334-8.832)	0.517
Other Governmental	0.357 (0.202-0.630)	<0.001	1.557 (0.673-3.604)	0.301	1.369 (0.244-7.675)	0.721
Unknown	0.704 (0.426-1.162)	0.170	1.582 (0.720-3.480)	0.254	1.217 (0.301-4.919)	0.783
Median Income						
< $38,000	*Reference		*Reference		*Reference	
$38,000-47,999	1.102 (0.839-1.449)	0.485	0.912 (0.656-1.269)	0.585	0.662 (0.368-1.193)	0.170
$48,000-62,999	1.147 (0.858-1.534)	0.354	0.993 (0.695-1.419)	0.970	0.411 (0.204-0.827)	0.013
>$63,000	0.864 (0.624-1.196)	0.379	1.004 (0.671-1.503)	0.986	0.507 (0.225-1.141)	0.101
Percent No High School Completion						
>21%	*Reference		*Reference		*Reference	
13-20.9%	0.953 (0.739-1.229)	0.710	0.930 (0.672-1.286)	0.660	1.316 (0.730-2.374)	0.361
7-12.9%	1.185 (0.907-1.548)	0.214	0.819 (0.572-1.175)	0.278	1.109 (0.556-2.212)	0.769
<7%	1.230 (0.897-1.686)	0.199	0.776 (0.514-1.170)	0.226	1.224 (0.522-2.869)	0.643
Urban/Rural Status						
Metro	*Reference		*Reference		*Reference	
Urban	1.277 (1.002-1.628)	0.048	1.147 (0,853-1.542)	0.363	1.837 (1.038-3.253)	0.037
Rural	2.648 (1.516-4.626)	<0.001	1.080 (0.552-2.113)	0.823	0.676 (0.145-3.153)	0.618
Great-Circle Distance						
0-24 miles	*Reference		*Reference		*Reference	
25-49 miles	0.959 (0.780-1.179)	0.690	0.942 (0.720-1.232)	0.663	0.748 (0.418-1.339)	0.329
50-99 miles	1.114 (0.852-1.457)	0.431	1.166 (0.863-1.574)	0.317	0.950 (0.504-1.792)	0.875
100+ miles	0.877 (0.680-1.132)	0.314	1.314 (0.976-1.768)	0.072	0.718 (0.357-1.447)	0.354
Stage						
Stage I	*Reference		*Reference		*Reference	
Stage II	1.232 (0.821-1.850)	0.314	1.053 (0.734-1.512)	0.779	0.927 (0.487-1.764)	0.817
Stage III	2.050 (1.150-3.655)	0.015	2.375 (1.324-4.258)	0.004	1.092 (0.302-3.944)	0.894
Stage IV	2.488 (1.700-3.643)	<0.001	2.231 (1.590-3.131)	<0.001	1.824 (0.933-3.565)	0.079
Unknown	1.815 (1.229-2.680)	0.003	1.715 (1.200-2.452)	0.003	0.910 (0.482-1.718)	0.771
Charlson-Deyo Score (Comorbidities)						
0	*Reference		*Reference		*Reference	
1	0.596 (0.410-0.868)	0.007	1.063 (0.668-1.690)	0.798	1.809 (0.772-4.240)	0.172
2	0.870 (0.481-1.574)	0.646	1.433 (0.544-3.772)	0.467	1.138 (0.150-8.608)	0.901
3	1.384 (0.497-3.851)	0.534	**		**	

## Discussion

Prior studies have established that mortality increases with metastasis at the time of diagnosis, a more proximal location of the primary tumor site, increased primary tumor size, advanced patient age, and increased LDH levels [1]. To the authors’ best knowledge, this study is the first one conducted to evaluate socioeconomic and geographical influences on patient prognosis in Ewing sarcoma. This study evaluated 3,238 patients diagnosed with Ewing sarcoma between 2004 and 2017 via univariate Kaplan-Meier curves and 1,267 patients via multivariate Cox regression and found that mortality at two years increased with advancing age, Black race, uninsured or Medicare insurance status, urban or rural residence location versus metropolitan, and primary tumors of stage III, IV, or unknown stage. At five years, mortality increased in patients aged 11-50 years and with primary masses of stage III, IV, or unknown stage. At all periods of follow-up, there was a significant increase in mortality in patients aged 31-40 years compared to patients aged 0-10. 

Prior studies have reported that mortality is increased in patients aged 18 or older [1]. This study further broke down mortality by age and found similar results for mortality at two and five years but not at ten years, except for patients aged 31-40 years. This discrepancy at ten years may be due to the low census of patients aged ≥41 years that were identified in this study, as more than 90% of patients were 0-40 years of age. Additionally, this study found that patients with tumors of higher stage had significantly increased mortality at two and five years, which was like other studies that reported metastasis as a poor prognostic factor [1]. Like other studies on other tumor types, such as osteosarcoma [8], this study found that mortality increased at two years for patients that were uninsured or utilizing Medicare as their primary payor at the time of diagnosis.

The effects of socioeconomic and geographic factors on mortality have a complex but important role in the outcomes of patients with cancer. Some of the factors identified in this study as being associated with increased mortality may have complex etiologies. Black race patients, for example, had increased mortality at two years but similar hazard ratios afterward, which may indicate that some Black patients are diagnosed at more advanced stages than White patients. This is potentially due to various socioeconomic disparities faced by minorities or because Ewing sarcoma is recognized as primarily affecting patients of European ancestry [1]. Increased two-year mortality for patients living in urban or rural areas may be due to reduced access to care and/or a lower quality of care in those regions when compared to metropolitan areas, which often have large and high-volume hospitals that are more equipped to readily identify, correctly diagnose, and effectively treat rare tumors such as Ewing sarcoma. Medicare use and uninsured status at the time of diagnosis may be associated with increased mortality as patients on Medicare are generally older and/or have more health issues, while uninsured patients may face difficulties when trying to obtain or pay for healthcare.

The goal of this study was to evaluate the effects of socioeconomic and geographical factors on the outcomes of patients diagnosed with Ewing sarcoma. In addition to discovering similar findings regarding age, tumor stage, and insurance status to those reported in previous studies, this study also identified increased two-year mortality in patients of Black race, patients living in rural or urban regions, and patients who are uninsured or utilizing Medicare. While metastasis at the time of diagnosis and tumor size may be recognized as some of the most important prognostic factors, socioeconomic and geographic factors certainly contribute to patient outcomes and may through delayed diagnosis, result in greater tumor size or increased frequency of metastasis at the time of diagnosis. Clinicians should be aware of these factors and address patients as individuals not only with unique biology but also unique difficulties when it comes to accessing or utilizing healthcare. Future studies may look at this topic more in-depth and explore the reasons behind barriers to care in different populations. Overall, addressing these discrepancies goes beyond the abilities of individual physicians and should be addressed by the medical community as a whole as we work to improve the wellness of our patients and healthcare on a civil, societal, and governmental level.

Limitations

This study is limited due to its retrospective nature and use of a large de-identified patient database. Reporting to the NCDB lacks uniform, standard patient follow-up intervals, and there is no long-term data. Mortality is reported for all causes and is not cancer-specific. There is selection bias in this study as the database includes cases only from accredited CoC hospitals. Not all data points were collected for all cases of Ewing sarcoma and, as a result, cases had to be excluded. Additionally, due to the low census of patients and median follow-up duration of 41 months, analysis and conclusions regarding outcomes may be limited.

## Conclusions

The most important previously recognized prognostic factors for patients with Ewing sarcoma are metastasis at the time of diagnosis, followed by primary site, size of the primary lesion, patient age, and LDH levels. This study contributed to the understanding of patient outcomes in Ewing sarcoma patients by identifying that mortality is increased in the first two years for patients of Black race, those living in urban or rural areas, and patients that are uninsured or using Medicare as their primary payor at time of diagnosis. It is critical for clinicians to recognize the unique barriers patients face when working to obtain medical care. Clinicians should strive to work with patients as people with unique biology and challenges so that they may cooperate to improve their individual health and the health of their community.
